# Pre-hospital glycemia as a biomarker for in-hospital all-cause mortality in diabetic patients - a pilot study

**DOI:** 10.1186/s12933-024-02245-8

**Published:** 2024-05-03

**Authors:** Salvatore Greco, Alessandro Salatiello, Francesco De Motoli, Antonio Giovine, Martina Veronese, Maria Grazia Cupido, Emma Pedarzani, Giorgia Valpiani, Angelina Passaro

**Affiliations:** 1 Department of Translational Medicine and for Romagna, University of Ferrara, Via Luigi Borsari, 46, 46 - 44121 Ferrara, Ferrara, Italy; 2 Medical Department, Azienda Unità Sanitaria Locale di Ferrara, Delta Hospital, Via Valle Oppio, 2, 44023 Lagosanto, Ferrara, Italy; 3 Department of Computer Science, University of Tübingen, Geschwister-Scholl-Platz, 72074 Tübingen, Germany; 4 Local Health Unit of Ferrara, Medical Direction, Via Cassoli, 30, 44121 Ferrara, Italy; 5 Research and Innovation Unit, Azienda-Ospedaliero Universitaria di Ferrara, Via Aldo Moro, 8, 44124 Cona, Ferrara, Italy; 6 Long-term Care, Azienda Unità Sanitaria Locale di Ferrara, Delta Hospital, Via Valle Oppio, 2, 44023 Lagosanto, Ferrara, Italy; 7 Medical Dapartment, Azienda-Ospedaliero Universitaria di Ferrara, Via Aldo Moro, 8, 44124 Cona, Ferrara, Italy

**Keywords:** Type 2 diabetes mellitus, Glycemic variability, Glucose metabolism disorder, AdaBoost-FAS, Machine learning

## Abstract

**Background:**

Type 2 Diabetes Mellitus (T2DM) presents a significant healthcare challenge, with considerable economic ramifications. While blood glucose management and long-term metabolic target setting for home care and outpatient treatment follow established procedures, the approach for short-term targets during hospitalization varies due to a lack of clinical consensus. Our study aims to elucidate the impact of pre-hospitalization and intra-hospitalization glycemic indexes on in-hospital survival rates in individuals with T2DM, addressing this notable gap in the current literature.

**Methods:**

In this pilot study involving 120 hospitalized diabetic patients, we used advanced machine learning and classical statistical methods to identify variables for predicting hospitalization outcomes. We first developed a 30-day mortality risk classifier leveraging AdaBoost-FAS, a state-of-the-art ensemble machine learning method for tabular data. We then analyzed the feature relevance to identify the key predictive variables among the glycemic and routine clinical variables the model bases its predictions on. Next, we conducted detailed statistical analyses to shed light on the relationship between such variables and mortality risk. Finally, based on such analyses, we introduced a novel index, the ratio of intra-hospital glycemic variability to pre-hospitalization glycemic mean, to better characterize and stratify the diabetic population.

**Results:**

Our findings underscore the importance of personalized approaches to glycemic management during hospitalization. The introduced index, alongside advanced predictive modeling, provides valuable insights for optimizing patient care. In particular, together with in-hospital glycemic variability, it is able to discriminate between patients with higher and lower mortality rates, highlighting the importance of tightly controlling not only pre-hospital but also in-hospital glycemic levels.

**Conclusions:**

Despite the pilot nature and modest sample size, this study marks the beginning of exploration into personalized glycemic control for hospitalized patients with T2DM. Pre-hospital blood glucose levels and related variables derived from it can serve as biomarkers for all-cause mortality during hospitalization.

**Supplementary Information:**

The online version contains supplementary material available at 10.1186/s12933-024-02245-8.

## Introduction

Diabetes mellitus is a group of metabolic disorders characterized by hyperglycemia caused by insufficient insulin secretion, weak peripheral insulin action, or both. It is known for being among the most prevalent human conditions and for its strong association with increased cardiovascular risk [1]: in fact, it is able to act as an independent risk factor for several cardiovascular diseases in both males and females [2] and predisposes the diabetic subjects to chronic micro- and macro-vascular complications [3].

Among the conditions within the diabetes spectrum, Type 2 diabetes mellitus (T2DM) is experiencing a growing prevalence worldwide because of the aging of the population and the increasing prevalence of a sedentary lifestyle and obesity: in Italy, it has reached a prevalence of 5.9%, affecting 3.5 million individuals (data from ISTAT for the year 2020, source: www.istat.it) with a similar prevalence among both males and females.

The comprehensive impact of diabetes poses a substantial economic and social challenge. In 2018, the Italian National Health Service (Servizio Sanitario Nazionale, SSN) allocated approximately €113.6 billion for public healthcare expenditure (source: “Monitoraggio della spesa sanitaria MEF– Report 2019”). According to official reports, a staggering €9.5 billion was needed exclusively to address the expenses related to diabetes treatment, constituting a significant 8.3% of the total healthcare expenditure.

In terms of hospital admissions, in 2014, Italy witnessed 17% of diabetics requiring at least one hospitalization, of which 5% were treated in a day hospital setting (source: https://www.siditalia.it/ricerca/centro-studi-e-ricerche/22-ricerca/centro-studi-e-ricerche/68-arno-diabete). These statistics closely mirror those commonly observed in the United States [4] in terms of hospital admissions for diabetic patients.

Compounding the situation, individuals with diabetes face an increased likelihood of experiencing comorbidities affecting other organs compared to non-diabetic individuals. In such instances, treatment often necessitates prolonged hospital stays and is associated with an unfavourable prognosis [5].

Among the main ramifications of hospitalization for patients with T2DM, the risk of experiencing hypo- and hyperglycemic events is exceptionally high and can have dramatic consequences [4–6]. Factors contributing to hypoglycemia are diverse, including: patients’ inability to balance food and medications appropriately; the need for insulin treatment and the suspension of home therapies with possible medication overdoses; metabolic imbalances resulting from acute events; inadequate nutritional support in response to acute events, known for being extremely common among hospitalized patients in various care settings [7, 8].

Hyperglycemic events are also frequently observed in diabetic patients upon hospital admission and during their stay, typically in conjunction with acute events, which usually involve internal medical conditions, injuries, burns, and surgical procedures [9, 10]. In particular, the prevalence of patients with blood glucose levels higher than 140 mg/dL upon hospital admission ranges from 32 to 40% (this data includes both previously known diabetic patients and individuals without a history of the disease) [11]. The latter group encompasses patients with undiagnosed or newly onset diabetes and individuals with stress-induced hyperglycemia [12]: this is a transient condition that appears during an acute illness and disappears upon its resolution, without subsequent evidence of diabetes mellitus: measuring glycated hemoglobin (HbA_1c_) can help discriminate this particular subgroup of patients.

It is crucial to emphasize that irrespective of prior T2DM diagnoses, the presence of in-hospital hyperglycemia is associated with an elevated risk of morbidity and mortality during the hospitalization period. Additionally, this condition contributes to the economic burden by extending hospital stays [9, 10].

While the literature is quite unanimous in stating that adequate at-home glycemic control can prevent long-term complications of diabetes and a significant portion of related hospitalizations [13], the therapeutic management of diabetes during hospitalization is different and, to some extent, more controversial [14]. Indeed, there is a notable discrepancy in terms of scientific evidence between how a diabetic individual is managed at home versus a diabetic patient in a hospital setting.

The primary goal of healthcare providers toward diabetic patients has always been to minimize the patient’s metabolic misalignment, primarily by preventing hypoglycemic events and excessive metabolic swings during hospitalization. It has long been known that overly tight glucose control, particularly in critically ill patients, can lead to severe hypoglycemia, causing discomfort for the patient and adverse clinical outcomes [15].

Regarding the measurement of HbA_1c_ levels, current guidelines (American Diabetes Association, 2022) recommend its evaluation upon hospital admission for diabetic individuals who have not had it measured in the preceding three months. This recommendation also applies to non-diabetic patients who show fasting blood glucose alterations during their hospital stay (as previously stated) and offers several advantages for the healthcare provider: on one hand, they can assess whether the patient they are caring for was or was not at their target glycemic levels with home-based therapies; on the other hand, they can choose the most suitable treatment for the post-hospitalization period [16, 17].

Furthermore, most studies confirm the existence of a strong correlation between HbA_1c_ levels and the average blood glucose (over the past four months), even though the linearity of this relationship has been questioned by other mathematical models [18]. Nevertheless, under this assumption, it is possible to convert HbA_1c_ levels fairly accurately into average blood glucose levels, always referring to the last 120 days of the patient’s life.

As of today, there are no articles in the scientific literature that describe the relationship between pre-hospitalization and intra-hospitalization average blood glucose levels. To fill this gap, in this pilot study, we investigate the relationship between these two factors, and assess how they relate to 30-day all-cause mortality in our cohort of hospitalized diabetic patients. Furthermore, by leveraging a larger set of routine clinical variables collected during hospitalization, we developed a predictive Machine Learning (ML) model to identify the predisposition of hospitalized diabetic individuals to experience adverse clinical outcomes within 30 days of hospital admission.

## Materials and methods

### Study design and participants

The study is of a pilot, observational, retrospective, monocentric type. It involves T2DM subjects hospitalized at the Internal Medicine Department of Delta Hospital in Lagosanto (Ferrara), between October 2022 and July 2023. These subjects were consecutively enrolled according to pre-established inclusion and exclusion criteria.

Inclusion criteria: age ≥ 18 years, pre-hospitalization diagnosis of T2DM, measurement of HbA_1c_ levels performed at the time of admission (within the first 48 hours), hospitalization for medical reasons, initiation of insulin therapy with a basal-bolus regimen upon admission and discontinuation of previous antidiabetic therapies, at least 12 consecutive pre-prandial blood glucose determinations, hospitalization duration of at least 48 hours.

Exclusion criteria: age < 18 years, diagnosis of type 1 diabetes or no diagnosis of diabetes at the time of admission, no measurement of HbA_1c_ levels performed within the first 48 hours, less than 12 blood glucose determinations or non-consecutive measurements, in-hospital antidiabetic treatment other than basal-bolus insulin regimen, total hospitalization time less than 48 hours.

### Data collection

The data regarding demographic characteristics, comorbidities as well as information regarding smoking habits and outpatient antidiabetic pharmacotherapy, were collected from the NBS hospital information system. The same platform provided information on HbA_1c_ levels (collected from blood samples within the first 48 h to minimize the error in pre-admission average blood glucose measurement) and in-hospital fasting blood glucose measurements for each patient using the point-of-care testing (POCT) method. Pre-admission average blood glucose levels were deduced from the linear relationship with circulating HbA_1c_ levels, as explained above.

In-hospital blood glucose controls were performed according to the hospital’s protocol, pre-prandially for the three main meals (breakfast, lunch and dinner) with the administration of lispro (rapid-acting) insulin and before bedtime, followed by glargine (long-acting) insulin administration. Therefore, each patient was on a basal-bolus insulin regimen, with individualized dosages set by the ward physicians. The glucometer used for glucose measurement was an Accu-check Inform 2 (F. Hoffmann-La Roche AG®, Basel, Switzerland).

To provide a more accurate assessment of the comorbidity burden of the enrolled subjects, the Charlson Comorbidity Index (CCI), deeply used in longitudinal studies, was calculated for each of them [19].

It is noteworthy that in the collection of the data, there were no missing values. The flowchart recapitulating the design of this study can be found in the Supplementary Fig. 1.

The study was conducted in full compliance with the principles of the Helsinki Declaration, and for its execution, specific guidelines for observational studies (STROBE - Strengthening the Reporting of Observational studies in Epidemiology) were followed [20].

### Statistical analysis

The data analyses were conducted using the software SPSS 29.0 (IBM SPSS Statistics, IBM Corporation) and MATLAB (MATLAB 2020a, The MathWorks, Natick, MA, USA).

Normal distribution of continuous variables was assessed using the Kolmogorov-Smirnov and Shapiro-Wilk tests. Categorical variables were summarized using frequencies and percentages, while continuous data were presented as mean ± standard deviation (SD). The Mann-Whitney U test (or Student’s t-test when necessary) was employed for continuous variables, and the χ2 test was used for categorical variables.

Based on the variables collected at the time of patient admission to the ward, as well as those related to in-hospital blood glucose levels, various ML models were utilized to seek the most accurate predictive model for 30-day mortality.

### Data preprocessing

From the initial dataset, invalid columns were removed (those containing anonymized data of enrolled subjects and information related to the length of hospital stay in days), as well as those containing constant values (specifically, the presence of a previous peptic ulcer and AIDS, which were not observed in our cohort).

Secondly, a normalization process was applied to the continuous variables, scaling them to have values between 0 and 1. The dataset was then randomly split into a training set and a test set, using 75% and 25% of the data, respectively. During the creation of these splits, a stratified random sampling was employed to ensure a similar fraction of surviving and deceased patients in both data divisions. This helps maintain a balanced representation of outcomes in the training and testing datasets. Further details about the characteristics of training and test data are provided in the appendix.

### Data augmentation

To deal with the large class imbalance (a significantly greater number of surviving patients compared to deceased patients), we performed class balancing by generating synthetic samples from the minority class using the SMOTE method [21]. These synthetic samples were used solely for model training but were not employed for evaluating the model’s performance. Additionally, it is worth noting that they are not required for the model’s use in clinical contexts.

### FAS (feature augmentation and selection)

Next, we introduced additional features by calculating squared terms (X^2^) for each continuous variable X, and interaction terms (Y*Z) for each pair of variables (Y, Z). As a final step, we performed feature selection by fitting a LASSO logistic regression model [22, 23]. In the remainder of this work, we will refer to this feature engineering procedure as Feature Augmentation and Selection (FAS).

### Machine learning models

This study used AdaBoost [24] with Feature Augmentation and Selection (AdaBoost-FAS) to construct a reliable classifier of 30-day mortality risk. This choice was motivated by the fact that AdaBoost is one of the most advanced methods for tabular data, such as those in this study. In brief, AdaBoost is a special type of ensemble method, which classifies data by computing weighted averages of the classification decisions of weaker base classifiers. In particular, the model used in this study uses decision stumps (single-level decision trees) as weak base classifiers.

For the purpose of model comparison, five additional models were considered: Majority, which is the majority classifier and always predicts the majority class in our cohort, that is, survival; LASSO-FAS, a LASSO logistic regression model with FAS; DecTree, a decision tree model; DecTree-FAS, a decision tree model with FAS; and AdaBoost, a boosting model with decision stumps without FAS, i.e., trained on the original features. Due to their lower predictive performance of 30-day mortality compared to the AdaBoost-FAS model, further details for these models will not be provided. We also provided further comparisons with other strong baseline classifiers in the appendix.

### Model reliance analysis

This analysis was conducted for the best-performing model, AdaBoost-FAS, with the goal of identifying the features on which the model relies the most for its predictions. The analysis is based on measuring the average increase in classification error when a feature is perturbed. Perturbations are applied by replacing the values of the target feature with random samples from the dataset while keeping the rest of the non-target features unchanged.

### SD and CV

Regarding the in-hospital blood glucose values, the mean standard deviation (SD) was calculated for the entire population. A Receiver Operating Characteristic (ROC) curve was plotted based on this SD, and an optimal cut-off value associated with 30-day mortality from the patient’s admission was chosen; the most appropriate cut-off value was found selecting the point on the ROC curve with the minimum distance from the left-upper corner of the unit square.

Similarly, the in-hospital glycemic coefficient of variation (CV) and the estimated pre-hospital glycemic coefficient of variation were calculated, and their respective cut-off values were determined. Based on these cut-offs, both for SD and CV (in-hospital and estimated pre-hospital), the diabetic population was divided into two groups (above and below the SD and CV values), and univariate comparative analyses were conducted among these groups.

Variables with a pvalue ≤ 0.05 in the univariate analyses were included in a multivariate logistic regression analysis to determine which of these variables was most closely related to the same outcomes. Odds Ratios (OR) and 95% confidence intervals (95% CI) were calculated for each variable. All pvalues ≤ 0.05 were considered statistically significant.

Lastly, Cox regression analyses were performed with adjustments for age, sex, and comorbidities to define the survival rates (and the corresponding survival curves) of various subgroups of diabetic patients based on SD, in-hospital CV, and estimated pre-hospital CV values above or below the predetermined cut-offs.

## Results

In this study, a total of 120 individuals with T2DM were enrolled and hospitalized at the Internal Medicine Department of Delta Hospital in Lagosanto (Ferrara) between October 2022 and July 2023.

**Fig. 1:**
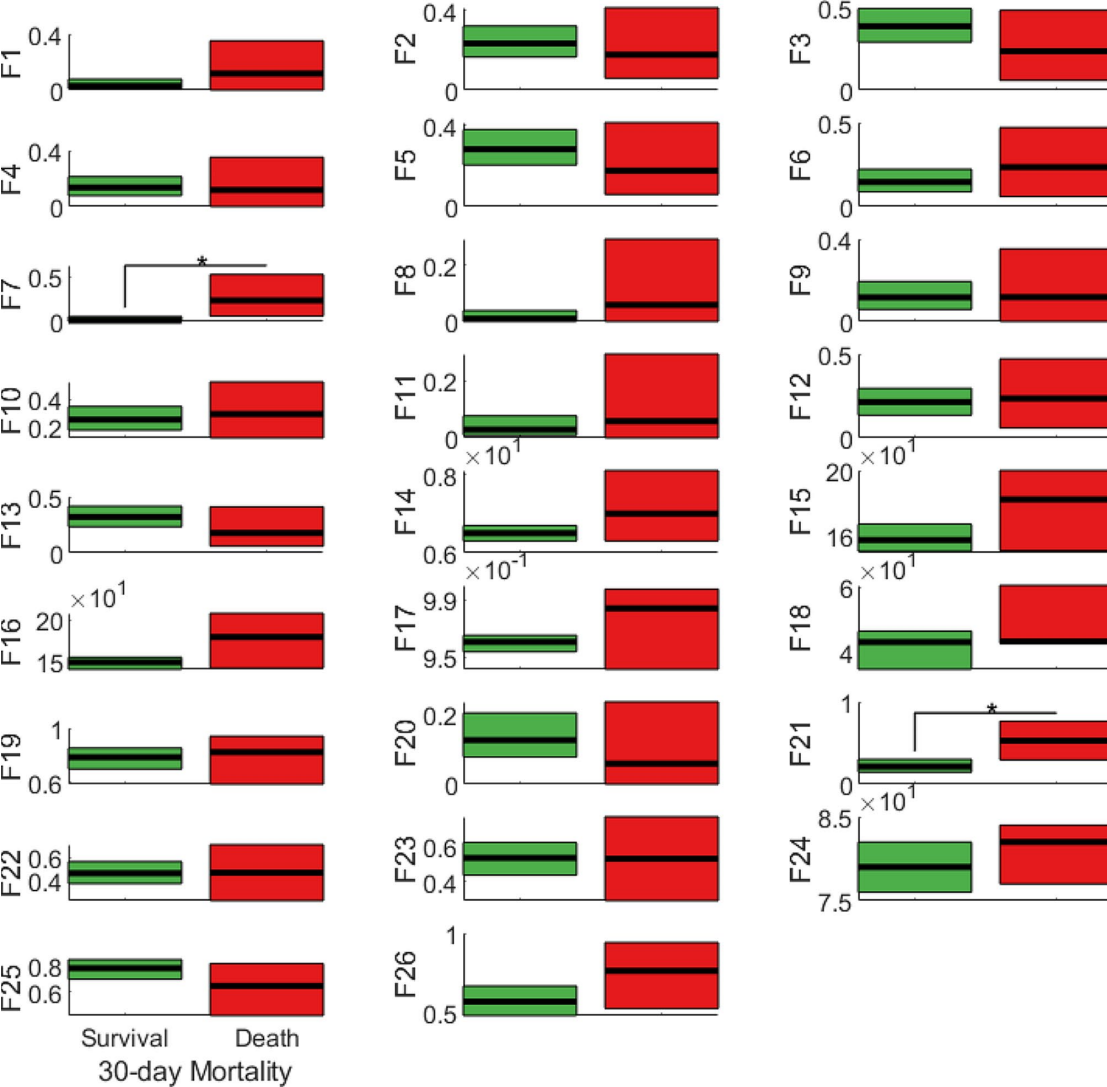
Comparative analysis between groups of patients who survived for 30 days (green boxes on the left) and those who passed away during the same period (red boxes on the right). F1, SGLT-2 inhibitor; F2, localized or hematological neoplasm; F3, smoking habit; F4, heart failure; F5, Chronic Kidney Disease (CKD); F6, stroke or Transient Ischemic Attack (TIA); F7, hemiplegia; F8, rheumatic disease; F9, dietary therapy; F10, metformin only; F11, DPP-4 inhibitors only; F12, insulin only; F13, other drugs/drug combinations; F14, HbA_1c_; F15, average in-hospital blood glucose; F16, median in-hospital blood glucose; F17, in-hospital/pre-hospital blood glucose ratio; F18, in-hospital glycemic variability (SD); F19, Hirsch’s compensation; F20, peripheral artery disease; F21, dementia; F22, uncomplicated T2DM; F23, complicated T2DM; F24, age; F25, hypertension; F26, sex

As mentioned, after recording the values of all the variables of interest in an electronic spreadsheet, non-parametric comparative analyses were conducted between groups of patients who survived and those who died within 30 days of admission using MATLAB software. Figure 1 succinctly presents the results of the non-parametric tests: the green and red boxes represent the CIs obtained for patients who survived and those who did not, within 30 days of hospital admission, respectively. The horizontal black lines represent the median values of the variable under consideration (for continuous variables) or the fraction of patients with that specific variable (for categorical variables), while asterisks indicate variables that exhibit a statistically significant difference between the two patient subgroups.

From the analyses, it appears that there are two variables that are significantly different between the subgroups of patients who survived and those who died within 30 days of hospital admission, namely, hemiplegia and dementia, which were present in the deceased population in significantly higher percentages.

**Fig. 2:**
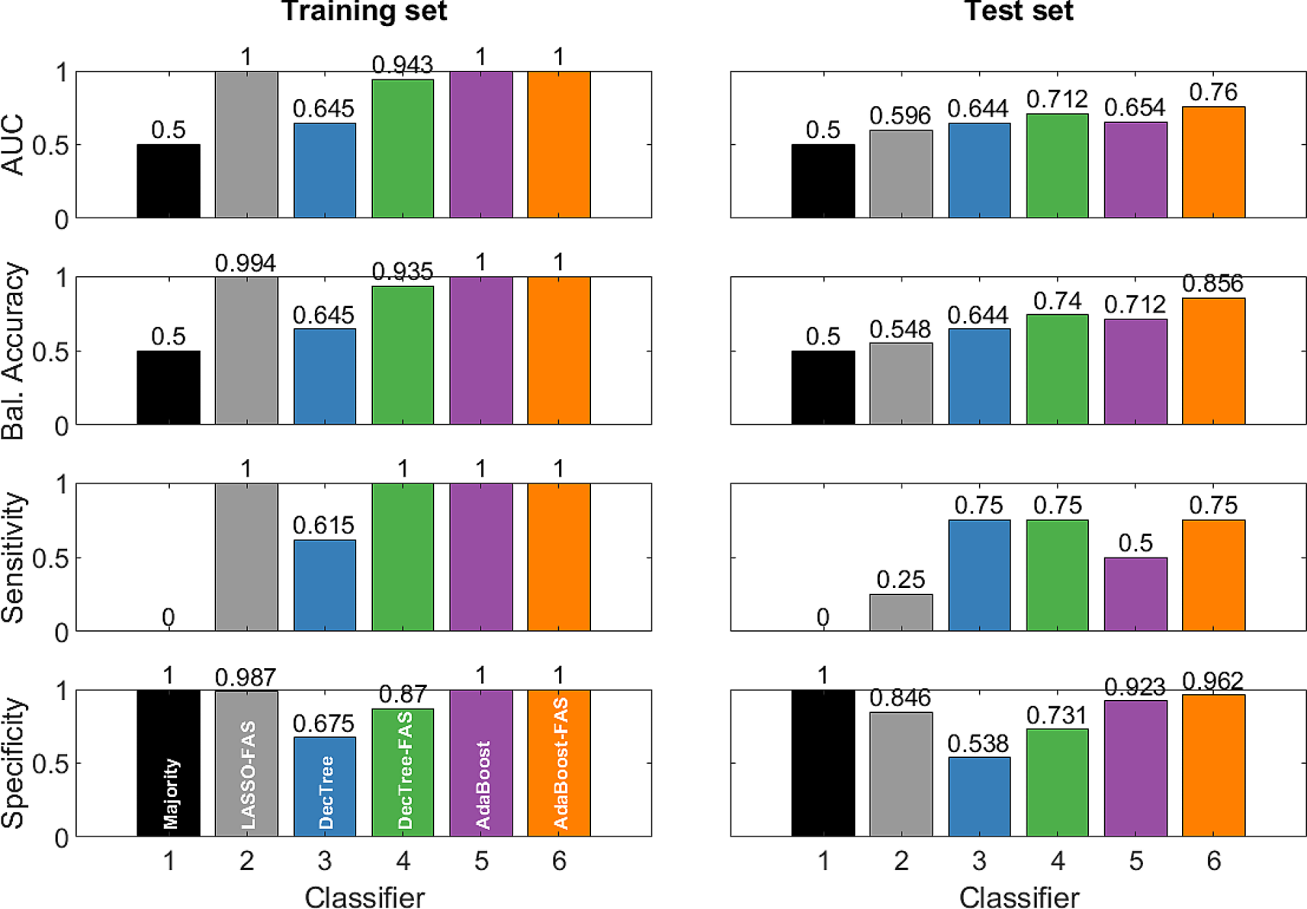
The different predictive models used for the analysis of the variables under study. AdaBoost-FAS (in orange) exhibits the overall best performance compared to all the others

Using ML techniques, as explained above, several predictive models for 30-day mortality were designed and trained. As expected, among these models, the AdaBoost-FAS classifier (depicted in orange in Fig. 2) exhibited the best performance: this model demonstrated high values of accuracy (0.856), sensitivity (0.75), and specificity (0.962), along with an Area Under Curve (AUC) of 0.76, indicating overall accurate predictive capabilities.

Importantly, the same model trained without FAS (AdaBoost) achieved inferior results in all considered metrics, underscoring the effectiveness of the FAS procedure. It is worth noting that FAS proved effective also for other models, like the considered decision tree. Also in this case, DecTree-FAS outperformed DecTree in all the considered metrics, making it the second-best model.

Taken together, these results demonstrate the superior performance of AdaBoost-FAS in predicting in predicting 30-day mortality compared to the other models considered and highlight the effectiveness of the FAS procedure in boosting classification performance.

The strength of AdaBoost-FAS (and AdaBoost) derives from its reliance on a set of *L* weak base classifiers, whose predictions are weighted according to:

$$G\left( x \right) = sign\left( {\sum\limits_{l = 1}^L {{\alpha _l}{g_l}\left( x \right)} } \right)$$

In the equation above, *x* is a vector collecting all the features associated with a patient, g_l_(x) is the prediction of the weak classifier with index *l*, α_l_ is the weight assigned to the prediction of the weak classifier *l*, and sign(•) represents the function that maps negative values to -1 (deceased individuals) and positive values to 1 (surviving individuals). Finally, *G(x)* represents the strong model that is used to classify patients. Note that the weights α_l_ determine how strongly the final predictions of *G(x)* are influenced by the weak classifiers g_l_(x).

**Fig. 3:**
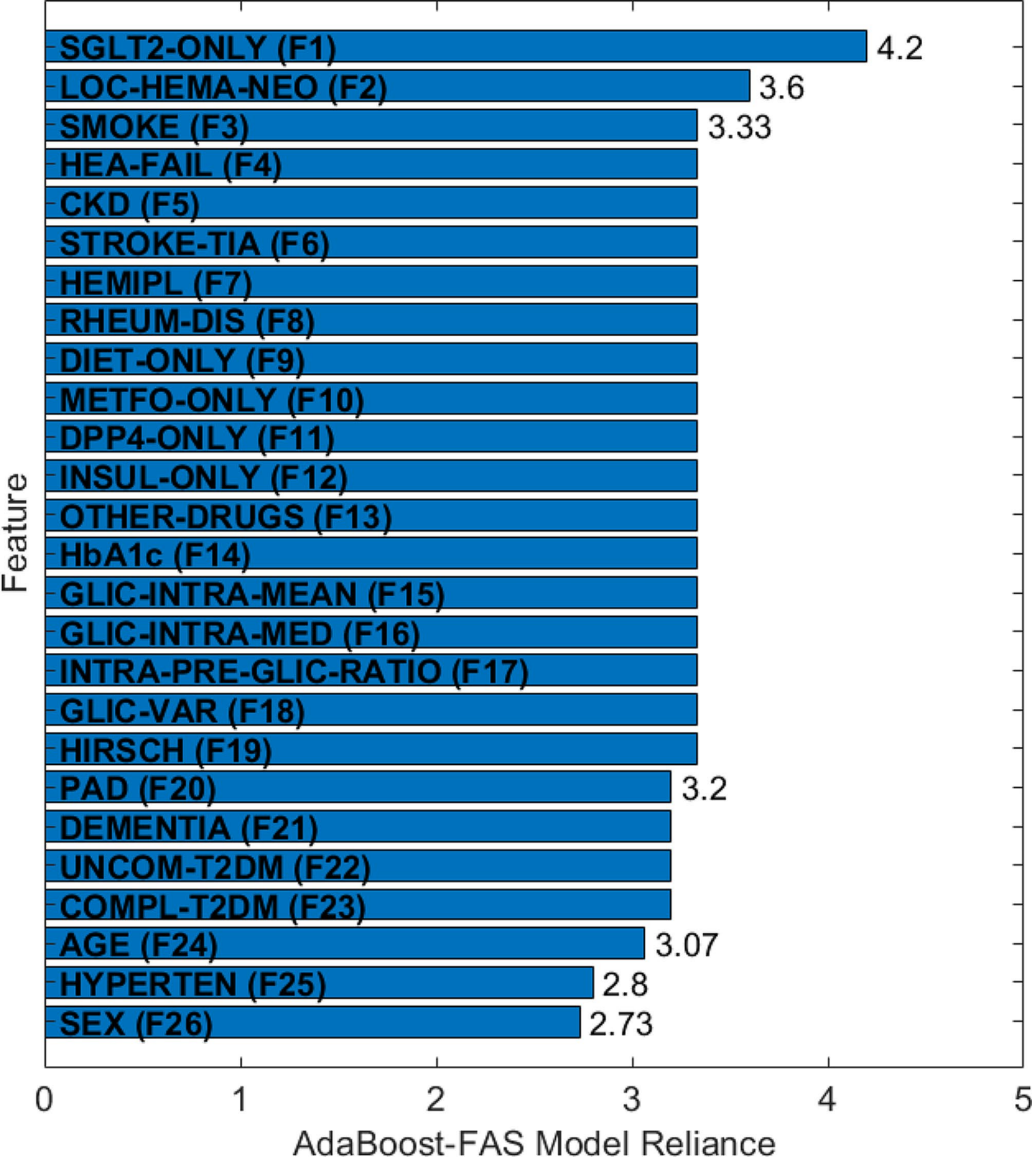
Model Reliance Analysis for AdaBoost-FAS. The variables involved, represented in an abbreviated manner, are the same as those described in Fig. 2

The model reliance analyses for AdaBoost-FAS are reported in Fig. 3. The figure shows that the most important variables for this model are exclusive home treatment with SGLT-2 inhibitors (reliance/dependency 4.2) and a positive history of non-metastatic hematological or solid neoplasms (reliance/dependency 3.6). This means that, in case of perturbation of these variables, the classification error of the entire model increases by a factor of 4.2 and 3.6 (for SGLT-2 inhibitors and non-metastatic hematological/solid neoplasms, respectively).

However, overall, the model’s reliance is similar for all the features, suggesting that none of them is more determinant than the others in steering the model’s predictions. Instead, it appears that all these features together are more or less equally critical for predicting 30-day mortality rate.

Due to the difficulty in finding a predictive model that is easy and immediately interpretable, especially from a clinician’s perspective, additional variables were explored to provide useful information within the context of 30-day mortality.

After defining population subgroups based on the death event within the 30^th^ day of hospital admission and the levels of SD and glycemic CV (in-hospital and estimated pre-glycemic, as explained above), descriptive analyses were conducted to identify significant differences among these subgroups.

First and foremost, the average age of the population was found to be 78.4 ± 8.9 years, demonstrating a predominantly elderly population, consistent with the average age of patients admitted to the Internal Medicine Departments of hospitals in the Ferrara provincial area; 60.0% of the patients were male, while 36.7% were smokers at the time of admission or had a history of smoking.

Details about the overall population, including comorbidities, average and median glycemic levels, glycemic variability (SD), and home-based antidiabetic therapies, can be found in Table 1 below.

A first stratification based on outcome (death yes/no) revealed substantial differences between subgroups of diabetic patients in terms of hospitalization duration in days (9.3 ± 4.6 vs. 15.5 ± 12.6 days, *p* = 0.033), with shorter average stays in the deceased population. Additionally, substantial differences were observed in the percentages of hemiplegia (23.5% vs. 1.0%, *p* < 0.001), a history of metastatic neoplasia (17.6% vs. 3.9%, *p* = 0.025), and dementia (52.9% vs. 21.4%, *p* = 0.006), all of which were significantly higher in non-surviving patients.

The population stratified by levels of glycemic variability (SD) using a cut-off value of 42.55 mg/dl showed differences in HbA_1c_ (6.3 ± 0.9% vs. 7.5 ± 1.9%, *p* = 0.018), average pre-hospitalization blood glucose (134.2 ± 27.1 vs. 168.2 ± 53.4 mg/dl, *p* = 0.017), average in-hospital blood glucose (138.2 ± 20.4 vs. 189.1 ± 39.8 mg/dl, *p* < 0.001), median in-hospital blood glucose (133.0 ± 20.4 vs. 158.9 ± 40.8 mg/dl, *p* < 0.001), and glycemic variability (29.2 ± 7.8 vs. 61.4 ± 16.1 mg/dl, *p* < 0.001). Additional differences were observed in the percentages of patients with a previous diagnosis of chronic obstructive pulmonary disease (COPD) (16.7% vs. 33.3%, *p* = 0.038) and home-based dietary therapy (18.5% vs. 6.1%, *p* = 0.034), as well as in the percentage of patients treated exclusively with insulin (11.1% vs. 30.3%, *p* = 0.011). In essence, this stratification revealed that patients with higher in-hospital glycemic variability not only had higher in-hospital blood glucose levels but also started with significantly higher blood glucose levels at home and were more frequently on insulin therapy (and vice versa much less frequently on dietary control).

As for the second stratification (for CV < or ≥ 25.8%), the two populations showed substantial differences in terms of hospitalization duration (11.3 ± 6.5 vs. 16.8 ± 14.2 days, *p* < 0.001), HbA_1c_ (6.5 ± 0.9% vs. 7.3 ± 1.9%, *p* = 0.038), average pre-hospitalization blood glucose (140.2 ± 27.0 vs. 161.4 ± 54.8 mg/dl, *p* = 0.036), average in-hospital blood glucose (150.5 ± 35.5 vs. 176.6 ± 41.6 mg/dl, *p* = 0.001), median in-hospital blood glucose (146.5 ± 34.0 vs. 167.2 ± 43.0 mg/dl, *p* = 0.006), and glycemic variability (30.0 ± 10.0 vs. 58.2 ± 18.1 mg/dl, *p* < 0.001). These subgroups also differed significantly in the percentage of patients with a COPD diagnosis (16.7% vs. 31.9%, *p* = 0.047), dietary therapy (18.8% vs. 6.9%, *p* = 0.047), and home insulin therapy (12.5% vs. 27.8%, *p* = 0.037). Patients with higher glycemic coefficients of variation thus had a longer average length of stay, along with higher average in-hospital and pre-hospitalization blood glucose levels.

The final stratification was based on a coefficient of variation that involved in-hospital glycemic variability (i.e. SD) and pre-hospitalization average blood glucose levels. This variable was termed “estimated pre-hospitalization glycemic coefficient of variation (CV)” and the ROC curve generated for it established a cut-off value of 28.8%. In this case, the two subpopulations showed significant differences in terms of median pre-hospitalization blood glucose (144.7 ± 35.2 vs. 171.3 ± 41.5 mg/dl, *p* < 0.001), glycemic variability (32.8 ± 13.8 vs. 59.3 ± 17.6 mg/dl, *p* < 0.001), and the ratio between average in-hospital and pre-hospitalization blood glucose (1.01 ± 0.17 vs. 1.21 ± 0.30, *p* = 0.005). Here, subjects with higher estimated pre-hospitalization CV values had higher median blood glucose levels and glycemic variability.

All the analyses just described are presented in Table 1. As for the data on 30-day mortality, significant differences between groups were observed exclusively among subjects with estimated pre-hospitalization CVs above or below the cut-off of 28.8%, with those below significantly advantaged over the others (7.1% of deceased subjects vs. 20.3%, *p* = 0.039). Although the raw numerical data is identical for all three columns (*n* = 4 vs. *n* = 13), the percentages and corresponding pvalues are different. The estimated pre-hospital CV is the only one able to achieve statistical significance (*p* = 0.039), demonstrating, at least in our patient cohort, a correlation between pre-hospitalization data and 30-day mortality. This analysis can be found in Table 2.

**Table 1:** Population descriptive analyses. 30-dDp, 30-day deceased patients; 30-dSp, 30-day survived patients; SD, standard deviation; CV, coefficient of variation; HbA_1c_, glycosylated haemoglobin; CKD, chronic kidney disease; TIA, transient ischemic attack; PAD, peripheral arterial disease; COPD, chronic obstructive pulmonary disease; T2DM, type 2 diabetes mellitus; DPP-4is, dipeptidyl peptidase 4 inhibitors; GLP-1 RAs, glucagon-like peptide 1 receptor agonists; SGLT-2is, sodium-glucose co-transporter 2 inhibitors

Personal informations	All patients (*n* = 120)	30-dDp (*n* = 17)	30-dSp (*n* = 103)	*p* value	SD < 42.55 (*n* = 54)	SD ≥ 42.55 (*n* = 66)	*p* value	In-hospital CV < 25.8% (*n* = 48)	In-hospital CV ≥ 25.8% (*n* = 72)	*p* value	Estimated Pre-hospital CV < 28.8% (*n* = 56)	Pre-hospital CV ≥ 28.8 (*n* = 64)	*p* value
Age, years ± SD	78.4 ± 8.9	80.7 ± 7.7	78.0 ± 9.1	0.25	78.3 ± 8.2	78.5 ± 9.5	0.90	78.9 ± 8.1	78.1 ± 9.4	0.60	77.2 ± 9.5	79.5 ± 8.3	0.15
Days of stay, n	12.0 ± 2.3	9.3 ± 4.6	15.5 ± 12.6	0.033	14.8 ± 12.5	14.4 ± 11.7	0.93	11.3 ± 6.5	16.8 ± 14.2	< 0.001	13.8 ± 12.2	15.2 ± 11.8	0.79
Females, *n* (%)	48 (40.0)	4 (23.5)	44 (42.7)	0.13	33 (61.1)	39 (59.1)	0.82	19 (39.6)	29 (40.3)	0.55	21 (37.5)	27 (42.2)	0.37
Males, *n* (%)	72 (60.0)	13 (76.5)	59 (57.3)		21 (38.9)	27 (40.1)		29 (60.4)	43 (59.7)		35 (62.5)	37 (57.8)	
Smoke habit, *n* (%)	44 (36.7)	4 (23.5)	40 (38.8)	0.23	16 (29.6)	28 (42.4)	0.15	15 (31.3)	29 (40.3)	0.21	18 (32.1)	26 (40.6)	0.22
Glycaemic informations													
HbA_1c_ (%)	7.0 ± 1.6	7.2 ± 1.2	6.9 ± 1.7	0.98	6.3 ± 0.9	7.5 ± 1.9	0.018	6.5 ± 0.9	7.3 ± 1.9	0.038	7.1 ± 2.0	6.8 ± 1.2	0.09
pre-hospital blood glucose, mean ± SD	152.9 ± 46.7	159.6 ± 35.8	151.8 ± 48.4	0.98	134.2 ± 27.1	168.2 ± 53.4	0.017	140.2 ± 27.0	161.4 ± 54.8	0.036	157.0 ± 58.6	149.3 ± 33.2	0.09
in-hospital blood glucose, mean ± SD	166.1 ± 41.2	177.8 ± 37.5	164.2 ± 41.6	0.88	138.2 ± 20.4	189.1 ± 39.8	< 0.001	150.5 ± 35.5	176.6 ± 41.6	0.001	149.1 ± 35.0	181.1 ± 40.6	0.09
in-hospital median blood glucose, mean ± SD	158.9 ± 40.8	173.7 ± 38.4	156.4 ± 40.8	0.83	133.0 ± 20.4	158.9 ± 40.8	< 0.001	146.5 ± 34.0	167.2 ± 43.0	0.006	144.7 ± 35.2	171.3 ± 41.5	< 0.001
in-hospital/pre-hospital average blood glucose ratio, mean ± SD	1.12 ± 0.27	1.14 ± 0.18	1.11 ± 0.28	0.18	1.08 ± 0.26	1.15 ± 0.27	0.14	1.10 ± 0.21	1.13 ± 0.30	0.20	1.01 ± 0.17	1.21 ± 0.30	0.005
glycemic variability, mean ± SD	46.9 ± 20.7	51.0 ± 14.0	46.3 ± 21.6	0.38	29.2 ± 7.8	61.4 ± 16.1	< 0.001	30.0 ± 10.0	58.2 ± 18.1	< 0.001	32.8 ± 13.8	59.3 ± 17.6	< 0.001
Comorbidities													
Hypertension, *n* (%)	92 (76.7)	11 (64.7)	81 (78.6)	0.21	40 (74.1)	52 (78.8)	0.54	37 (77.1)	55 (76.4)	0.56	40 (71.4)	52 (81.3)	0.15
Ischemic heart disease, *n* (%)	28 (23.3)	4 (23.5)	24 (23.3)	0.98	12 (22.2)	16 (24.2)	0.80	12 (25.0)	16 (22.2)	0.44	16 (28.6)	12 (18.8)	0.15
Heart failure, *n* (%)	16 (13.3)	2 (11.8)	14 (13.6)	0.84	6 (11.1)	10 (15.2)	0.52	5 (10.4)	11 (15.3)	0.32	5 (8.9)	11 (17.2)	0.15
Moderate or severe CKD, *n* (%)	32 (26.7)	3 (17.6)	29 (28.2)	0.36	17 (31.5)	15 (22.8)	0.28	14 (29.2)	18 (25.0)	0.38	15 (26.8)	17 (26.6)	0.57
Stroke or TIA, *n* (%)	19 (15.8)	4 (23.5)	15 (14.6)	0.35	7 (13.0)	12 (18.2)	0.44	7 (14.6)	12 (16.7)	0.48	9 (16.1)	10 (15.6)	0.57
PAD, *n* (%)	14 (11.7)	1 (5.9)	13 (12.6)	0.42	7 (13.0)	7 (10.6)	0.69	8 (16.7)	6 (8.3)	0.14	7 (12.5)	7 (10.9)	0.51
COPD, *n* (%)	31 (25.8)	3 (17.6)	28 (27.2)	0.41	9 (16.7)	22 (33.3)	0.038	8 (16.7)	23 (31.9)	0.047	11 (19.6)	20 (31.2)	0.11
Mild hepatopathy, *n* (%)	2 (1.7)	0 (0.0)	2 (1.9)	0.56	2 (3.7)	0 (0.0)	0.12	1 (2.1)	1 (1.4)	0.64	1 (1.8)	1 (1.6)	0.72
Severe hepatopathy, *n* (%)	1 (0.8)	0 (0.0)	1 (1.0)	0.68	1 (1.9)	0 (0.0)	0.27	0 (0.0)	1 (1.4)	0.60	0 (0.0)	1 (1.6)	0.53
Peptic ulcer disease, *n* (%)	0 (0.0)	0 (0.0)	0 (0.0)	-	0 (0.0)	0 (0.0)	-	0 (0.0)	0 (0.0)	-	0 (0.0)	0 (0.0)	-
AIDS, *n* (%)	0 (0.0)	0 (0.0)	0 (0.0)	-	0 (0.0)	0 (0.0)	-	0 (0.0)	0 (0.0)	-	0 (0.0)	0 (0.0)	-
Hemiplegia, *n* (%)	5 (4.2)	4 (23.5)	1 (1.0)	< 0.001	2 (3.7)	3 (4.5)	0.82	1 (2.1)	4 (5.6)	0.33	1 (1.8)	4 (6.3)	0.23
Localized or hematological malignancy, *n* (%)	27 (22.5)	3 (17.6)	24 (23.3)	0.61	11 (20.4)	16 (24.2)	0.61	8 (16.7)	19 (26.4)	0.15	11 (19.6)	16 (25.0)	0.32
Metastatic malignancy, *n* (%)	7 (5.8)	3 (17.6)	4 (3.9)	0.025	4 (7.4)	3 (4.5)	0.51	4 (8.3)	3 (4.2)	0.29	4 (7.1)	3 (4.7)	0.43
Dementia, *n* (%)	31 (25.8)	9 (52.9)	22 (21.4)	0.006	12 (22.2)	19 (28.8)	0.41	11 (22.9)	20 (27.8)	0.35	11 (19.6)	20 (31.3)	0.11
Rheumatological disease, *n* (%)	2 (8.3)	1 (5.9)	1 (1.0)	0.14	1 (1.9)	1 (1.5)	0.89	1 (2.1)	1 (1.4)	0.64	1 (1.8)	1 (1.6)	0.72
T2DM without chronic complications, *n* (%)	56 (46.7)	8 (47.1)	48 (46.6)	0.97	25 (46.3)	31 (47.0)	0.94	23 (47.9)	33 (45.8)	0.49	26 (46.4)	30 (46.9)	0.55
T2DM with chronic complications, *n* (%)	64 (53.3)	9 (52.9)	55 (53.4)		29 (53.7)	35 (53.0)		25 (52.1)	39 (54.2)		30 (53.7)	34 (53.1)	
CCI, mean ± SD	4.2 ± 2.4	5.2 ± 3.1	4.0 ± 2.2	0.06	4.2 ± 2.4	4.1 ± 2.4	0.94	4.1 ± 2.6	4.2 ± 2.3	0.76	4.0 ± 2.4	4.3 ± 2.4	0.53
Antidiabetic treatment													
Diet only, *n* (%)	14 (11.7)	2 (11.8)	12 (11.7)	0.99	10 (18.5)	4 (6.1)	0.034	9 (18.8)	5 (6.9)	0.047	8 (14.3)	6 (9.4)	0.29
Metformin only, *n* (%)	31 (25.8)	5 (29.4)	26 (25.2)	0.72	17 (31.5)	14 (21.2)	0.20	13 (27.1)	18 (25.0)	0.48	17 (30.4)	14 (21.9)	0.20
Sulphonylureas only, *n* (%)	2 (1.7)	0 (0.0)	2 (1.9)	0.56	1 (1.9)	1 (1.5)	0.89	0 (0.0)	2 (2.8)	0.36	1 (1.8)	1 (1.6)	0.72
DPP-4is only, *n* (%)	4 (3.3)	1 (5.9)	3 (2.9)	0.53	2 (3.7)	2 (3.0)	0.84	1 (2.1)	3 (4.2)	0.47	1 (1.8)	3 (4.7)	0.36
GLP-1 RAs only, *n* (%)	1 (0.8)	0 (0.0)	1 (1.0)	0.68	1 (1.9)	0 (0.0)	0.27	1 (2.1)	0 (0.0)	0.40	1 (1.8)	0 (0.0)	0.47
Insulin only, *n* (%)	26 (21.7)	4 (23.5)	22 (21.4)	0.84	6 (11.1)	20 (30.3)	0.011	6 (12.5)	20 (27.8)	0.037	11 (19.6)	15 (23.4)	0.39
SGLT-2is only, *n* (%)	5 (4.2)	2 (11.8)	3 (2.9)	0.09	2 (3.7)	3 (4.5)	0.82	2 (4.2)	3 (4.2)	0.67	2 (3.6)	3 (4.7)	0.56
Other drugs or combinations of drugs, *n* (%)	36 (30.0)	3 (17.6)	33 (32.0)	0.23	15 (27.8)	21 (31.8)	0.63	16 (33.3)	20 (27.8)	0.33	15 (26.8)	21 (32.8)	0.30

**Table 2:** 30-day mortality in the entire population, stratified by SD, in-Hospital CV, and estimated pre-hospital CV values An additional analytical phase was dedicated to logistic regressions to identify the factors majorly predisposing hospitalized patients to death within 30 days. Firstly, the predictive value of SD was tested (in this case, data pertains to levels ≥ 42.55 mg/dl, see Table 3 for details)

	Overall population (*n* = 120)	SD < 42.55 (*n* = 54)	SD ≥ 42.55 (*n* = 66)	*p* value	In-hospital CV < 25.8 (*n* = 48)	In-hospital CV ≥ 25.8 (*n* = 72)	*p* value	Estimated pre-hospital CV < 28.8 (*n* = 56)	Estimated pre-hospital CV ≥ 28.8 (*n* = 64)	*p* value
30-day mortality, *n* (%)	17 (13.7)	4 (7.4)	13 (19.7)	0.06	4 (8.3)	13 (18.1)	0.14	4 (7.1)	13 (20.3)	0.039

As previously observed in the univariate analyses, the presence of anamnestic variables such as hemiplegia and dementia proved to be extremely effective in predicting the negative outcome (OR 53.05, 95% CI 3.66-768.22, *p* = 0.004, and OR 5.36, 95% CI 1.23–23.36, *p* = 0.025, respectively). Following that, both sex (OR 7.64, 95% CI 1.25–46.74, *p* = 0.028) and the SD ≥ 42.55 mg/dl (OR 5.49, 95% CI 1.14–26.41, *p* = 0.034), reached statistical significance. Finally, the length of hospitalization also proved to be somewhat predictive of 30-day mortality (OR 0.89, 95% CI 0.78-1.00, *p* = 0.047). As seen in Table 1, patients who survived to the 30^th^ day also had longer average lengths of stay, indicating that most deaths among diabetic patients hospitalized in our department occur more often within the first 10 days of admission.

**Table 3:** Logistic regression for identifying variables associated with 30-day mortality, with the introduction of SD ≥ 42.55 mg/dl. SD, standard deviation; OR, odds ratio; CI, confidence interval

Variable	OR	95% CI (lower-upper)	*p* value
Age	1.06	0.97–1.17	0.22
Sex = M	7.64	1.25–46.74	0.028
SD ≥ 42.55 mg/dl	5.49	1.14–26.41	0.034
Days of stay	0.89	0.78-1.00	0.047
Hemiplegia	53.05	3.66-768.22	0.004
Metastatic malignancy	3.77	0.17–85.61	0.41
Dementia	5.36	1.23–23.36	0.025

**Table 4:** Logistic regressions for identifying variables associated with 30-day mortality, with the introduction of in-hospital glycemic CV ≥ 25.8%. SD, standard deviation; OR, odds ratio; CI, confidence interval

Variable	OR	95% CI (lower-upper)	*p* value
Age	1.08	0.97–1.19	0.15
Sex = M	8.64	1.23–60.84	0.030
In-Hospital CV ≥ 25.8%	7.53	1.25–45.58	0.028
Days of stay	0.86	0.75–0.99	0.030
Hemiplegia	33.01	2.64-411.49	0.007
Metastatic malignancy	3.94	0.12-126.99	0.44
Dementia	7.03	1.48–33.35	0.014

In the second logistic regression analysis (Table 4), instead of using values of SD ≥ 42.55 mg/dl, the in-hospital glycemic coefficient of variation (CV) ≥ 25.8% was introduced. This analysis confirmed the strong predictive value of hemiplegia (OR 33.01, 95% CI 2.64-411.49, *p* = 0.007) and dementia (OR 7.03, 95% CI 1.48–33.35, *p* = 0.014), while also highlighting the importance of an in-hospital glycemic CV ≥ 25.8% (OR 7.53, 95% CI 1.25–45.58, *p* = 0.028), of sex (in the case of male sex OR 8.64, 95% CI 1.23–60.84, *p* = 0.030), and of the length of hospital stay, just like in the previous analysis (OR 0.86, 95% CI 0.75–0.99, *p* = 0.030).

In the last logistic regression analysis, we introduced the estimated pre-hospital glycemic coefficient of variation (estimated pre-hospital CV ≥ 28.8%) with the same objectives set as before. Unsurprisingly, the factors most associated with 30-day mortality were hemiplegia (OR 51.32, 95% CI 3.70-711.21, *p* = 0.003) and dementia (OR 5.17, 95% CI 1.21–22.12, *p* = 0.027), the latter being equally significant as the sex variable (OR 9.48, 95% CI 1.28–69.98, *p* = 0.027). Following closely, we find the estimated pre-hospital CV ≥ 28.8% (OR 4.84, 95% CI 1.06–22.06, *p* = 0.042) and the length of hospitalization (OR 0.87, 95% CI 0.76-1.00, *p* = 0.046). Details of the above can be found in Table 5.

**Table 5:** Logistic regression for the identification of variables associated with 30-day mortality, with the introduction of the estimated pre-hospital CV ≥ 28.8%. SD, standard deviation; OR, odds ratio; CI, confidence interval

Variable	OR	95% CI (lower-upper)	*p* value
Age	1.06	0.96–1.17	0.23
Sex = M	9.48	1.28–69.98	0.027
Estimated pre-Hospital CV ≥ 28.8%	4.84	1.06–22.06	0.042
Days of stay	0.87	0.76-1.00	0.046
Hemiplegia	51.32	3.70-711.21	0.003
Metastatic malignancy	3.02	0.11–82.45	0.51
Dementia	5.17	1.21–22.12	0.027

Once the phase of logistic regression analysis was completed, Cox regression analyses, including survival functions, were conducted to understand the overall patient population’s outcomes up to the 30^th^ day of observation based on different classifications. As previously performed for logistic regression analyses, patients were stratified according to the levels of SD < or ≥ 42.55 mg/dl, intra-hospital glycemic CV < or ≥ 25.8%, and estimated pre-hospital glycemic CV < or ≥ 28.8%.

**Fig. 4:**
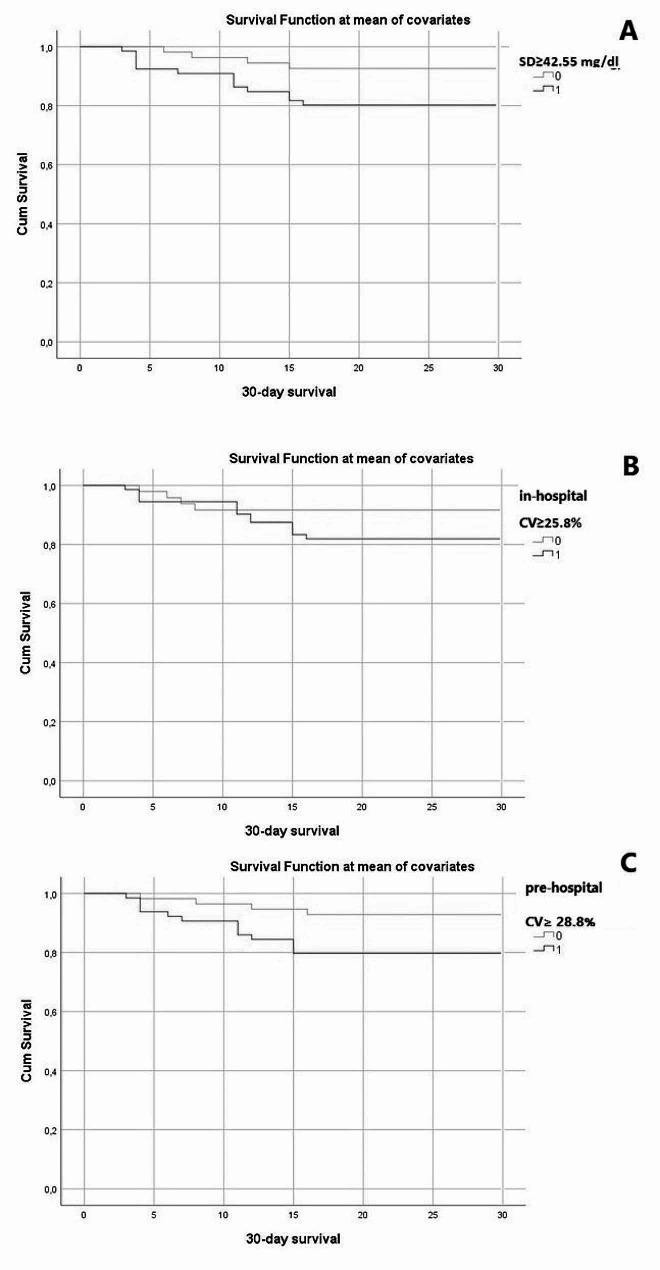
Cox Regression Analysis. Stratification of the diabetic population based on SD ≥ 42.55 mg/dl (box A), intra-hospital glycemic CV ≥ 25.8% (box B) and estimated pre-hospital glycemic CV ≥ 28.8% (box C). In red, the subgroup of patients with levels exceeding the cut-off; in blue, those with levels below the cut-off

In this case, adjustments were made for age, sex, and comorbidity burden (using the CCI) to reduce potential confounding factors. The survival curves derived from these three variables are depicted in Fig. 4 and indicate that patients with high levels of intra-hospital glycemic variability (≥ 42.55 mg/dl, red curve box A) and a high estimated pre-hospital glycemic CV (≥ 28.8%, red curve box C) more frequently experience an adverse outcome within the first 30 days of hospital admission. This underlines the significance of these variables in predicting 30-day mortality.

## Discussion

In recent years, scientific research has significantly expanded knowledge in the field of diabetology. Substantial changes in therapeutic practices have been coupled with significant improvements in the monitoring of diabetic individuals.

Compared to traditional measurements, which primarily focus on individual blood glucose values rather than the overall glycemic trends over a defined time frame, alternative glycemic profiles have gained prominence. These include “Time In Range” (TIR), which is associated with “Time Above Range” (TAR) and “Time Below Range” (TBR), representing the time period during which diabetic individuals maintain blood glucose values within predetermined normal ranges [24]. Other methods include the “Ambulatory Glucose Profile” (AGP), which involves continuous glucose monitoring for 24 h over a specified period (typically two weeks), glycemic variability, which tracks the frequency and amplitude of glycemic fluctuations, and the coefficient of glycemic variation (CV), which correlates the standard deviation (SD) of measured blood glucose values with the mean blood glucose level [25]. Among these methods, glycemic variability and CV undoubtedly offer a more effective assessment of the “glycemic health” of individuals with T2DM.

Back in 1970, Service et al. associated the concept of glycemic variability with glycemic excursions typically induced by meals, introducing the concept of the Mean Amplitude of Glycemic Excursions (MAGE), using glycemic excursions exceeding a single standard deviation (SD) above the mean glucose level [26]. However, it was only in 2003, thanks to a paper by Kovatchev et al., that the link between increased hypoglycemic events and high glycemic variability was established in both type 1 and T2DM [27].

In 2005, Hirsch, who developed a formula based on SD and mean glucose levels to assess if a series of glucose values followed good glycemic control, and Brownlee hypothesized that inconsistent glycemic control with high glycemic variability underlies the development of microvascular complications in diabetes, more so than chronic hyperglycemia [28]. In 2006, Monnier et al. suggested that these complications were more associated with high levels of oxygen free radicals induced not so much by chronic hyperglycemia but by large glycemic fluctuations [29]. In contrast, a few months later, Garg and colleagues demonstrated the significant benefits of continuous glucose monitoring (CGM) techniques, already within 10 days of use [30].

As mentioned above, less literature attention has been devoted to the relationship between the levels of average blood glucose sustained in the period preceding a hospital admission (in our case, for medical rather than surgical reasons) and in-hospital blood glucose levels. The approach to patients with T2DM during hospitalization is almost standardized, aiming to achieve a glycemic target that typically falls within the same ranges.

In most cases, oral antidiabetic therapies are suspended at hospital admission, and patients are transitioned to subcutaneous insulin administration using a basal-bolus regimen. There are exceptions for patients admitted to intensive care settings (where higher average glucose levels may be allowed due to acuity that benefits from more readily available resources), critically ill patients, and those considered more clinically stable: the latter group may be recommended to continue or resume home-based therapies also during hospitalization.

The primary objective of this work was to investigate the influence of pre-hospital admission blood glucose levels on the outcomes observed during the hospitalization period: intuitively, accessing these data is not straightforward, particularly when dealing with individuals who do not use continuous home monitoring techniques such as CGM or Flash Glucose Monitoring (FGM). One way to overcome this challenge is to use HbA_1c_ testing at the time of hospital admission, which is already strongly recommended by current guidelines. To obtain the estimated average pre-hospitalization blood glucose, a simple conversion table can be used, based on the linear correlation between HbA_1c_ levels and the average blood glucose [31].

An initial approach to characterize our patient population relied on the use of ML techniques to generate a predictive model of 30-day mortality from data collected during hospitalization. As shown in the previous sections, AdaBoost-FAS, the method we designed for our cohort, displayed impressive predictive abilities: for example, it was able to predict the 30-day mortality class of diabetic individuals with a balanced accuracy on the independent test set of 85.6%. The subsequent reliance analysis performed showed that, overall, the model is particularly sensitive to changes in two specific variables, namely the exclusive home treatment with SGLT-2 inhibitors (reliance = 4.2) and a positive history of non-metastatic hematological or solid neoplasms (reliance = 3.6). However, overall, the model predictions are also fairly sensitive to changes in 24 other variables (reliance > 2.7). This suggests that it is not possible to reliably determine the 30-day mortality risk based on measuring only a handful of variables. Therefore, providing an intuitive explanation of the decision-making process of the model to clinicians appears challenging and the potential lack of transparency may limit the trust clinicians place in the model, subsequently reducing the likelihood of its adoption.

To further discriminate the factors most associated with 30-day mortality in our patients, we decided to use classical statistics, including population descriptive analyses, logistic regressions, and survival curves, stratifying the population by outcome (death yes/no), levels of glycemic variability (SD < or ≥ 42.55 mg/dl), glycemic CV (< or ≥ 25.8%), and estimated pre-hospitalization glycemic CV (< or ≥ 28.8%). This last variable is not found in the literature and has been introduced by our research group: it consists of a ratio between glycemic variability recorded for our patients during hospitalization (expressed in mg/dl) and the pre-hospitalization mean glucose calculated from the HbA_1c_ levels of diabetic individuals, tested necessarily within the first 48 hours since hospital admission (also this last variable is expressed in mg/dl).

The rationale behind this calculation is to be found in the original purpose of the study, which is precisely to investigate the existence of a relationship between home blood sugar levels and those recorded during hospitalization. The fact that the numerator and denominator are expressed in the same unit of measurement also reduces the risk of over- or underestimating the value of individual variables.

From the analysis of our population, it emerged that there is a significant difference in the percentage of diagnoses of dementia or hemiplegia between patients who died within the first 30 days of hospitalization and those who survived. Patients with worse outcomes often had at least one of the diagnoses of dementia and hemiplegia upon admission to our department, and these factors have proven to be the most reliable in predicting 30-day mortality, even in logistic regression analyses. This is in line with current literature, and in 2022, a group of researchers from our university demonstrated on a dataset of over 3 million patients admitted to Italian Geriatrics and Internal Medicine Units that dementia is among the diagnoses most predisposing to in-hospital mortality [32]. The same occurs in the case of hemiplegia, an outcome in our patients often following a prior stroke, which, in turn, is a negative prognostic factor for diabetic and non-medically hospitalized subjects for all causes [33, 34].

The explanation behind this phenomenon is intuitively connected to the initial clinical conditions of patients with dementia or residual hemiplegia, who are considered intrinsically fragile and more predisposed to further acute events, such as additional cerebrovascular events, infections, or new cancer diagnoses. Therefore, it is the performance status of these subjects, combined with a limited organic response to acute events, that leads to an exitus in the short term of hospitalization.

Also, from population descriptive analyses, it emerged that both the subgroups of patients who died/survived within 30 days of hospitalization and those with intra-hospital blood glucose CVs greater or less than the threshold value of 25.8%, showed significantly different lengths of stay. In particular, both the patients who survived to the 30^th^ day and those with higher glycemic CVs experienced longer hospital stays. This demonstrates that diabetic subjects encountered worse outcomes, especially in the early stages of hospitalization, while those with longer hospital stays more frequently experienced higher fluctuations in blood glucose, suggesting the difficulty on the part of the clinicians in achieving adequate glycemic control in patients with a diagnosis of T2DM.

Similarly, significant differences between groups (population stratified by SD and intra-hospital blood glucose CV) were found in terms of HbA_1c_ levels. Once again, higher levels of glycated hemoglobin (and therefore average pre-hospital blood glucose levels) were found in subjects who experienced greater blood glucose fluctuations during hospitalization (both SD and CV), further demonstrating that poor at-home glycemic control can (not only theoretically) predispose to equally inadequate in-hospital glycemic control.

The data related to blood glucose levels, which vary significantly between patient subgroups with different SDs and CVs both during and before hospitalization, are straightforward and therefore do not require further comments. It is worth noting that the “pure” data regarding mean and median blood glucose levels during and before hospitalization did not reach statistical significance in distinguishing between patients who died and those who survived on the 30^th^ day of hospitalization.

Regarding another noticeable difference in Table 1, namely the percentages of subjects with COPD in the subgroups stratified by SD and intra-hospital blood glucose CV, it can be hypothesized (although not proven, as no checks were conducted on medical treatments administered during hospitalization) that among the reasons for hospital admission for these patients, there may have been an illness needing treatment with steroid medications (such as exacerbations of chronic pulmonary diseases) and, therefore, a cause of significant increases in blood glucose levels during the hospital stay. This would be in line with current literature and would justify the inadequate glycemic controls observed in some subpopulations [35].

The subgroups of the population stratified by death/survival within 30 days also differed in terms of the medical history component related to metastatic tumoral disease, with nearly double percentages in the deceased population. This could be explained in the same way as described for the variables “hemiplegia” and “dementia,” although it cannot be denied that the small sample size (4 subjects per subgroup had the same variable) in this case may have overestimated the difference.

As for the differences in terms of at-home treatment, the data should be interpreted similarly to what was mentioned earlier regarding HbA_1c_; substantial differences between groups were observed for dietary therapy, which was more prevalent in the subgroup with lower SD and intra-hospital blood glucose CV, and insulin therapy, with the opposite trend. In this case as well, it can be hypothesized that patients treated with milder therapies, such as dietary therapy, and presumably accustomed to achieving lower blood glucose levels at home (unlike those treated with insulin), were somehow less prone to metabolic imbalances even during hospitalization.

Regarding the analysis shown in Table 2, there is not much to say: in our case, the estimated pre-hospital CV is the only variable to achieve a statistical significance in association with 30-day mortality and it demonstrates, at least in our patient cohort, a possible theoretical correlation between pre-hospital mean glycemia and hospitalization outcomes. Whether this will have a real-world counterpart remains to be fully demonstrated in other studies with larger sample sizes, since the introduction of the variable in the literature was made for the first time in this pilot study.

The logistic regression analyses have been partly discussed, at least regarding the predictive role of “hemiplegia” and “dementia” in 30-day mortality. In these analyses, age, sex (male sex was used as reference), and length of stay in days were always considered as potential variables determining better or worse outcomes. The first of the three regressions showed that, after hemiplegia and dementia, the variables sex and SD ≥ 42.55 mg/dl are capable of independently influencing 30-day mortality in our patients (OR 7.64 and 5.49, respectively). In both cases, the data is highly consistent with current literature: male sex is known to be the foremost unmodifiable risk factor for in-hospital mortality and predetermined time intervals after admission [36, 37]. Similarly, there are no particular doubts about the deleterious effects of high glycemic variability (expressed as SD) during hospitalization. A 2017 study by British researchers revealed a strong association between high glycemic variability and increased in-hospital mortality in a population of over 28,000 subjects [38], and a 2022 Korean study conducted on over 2,000 patients (diabetic and non-diabetic) admitted for acute heart failure showed increased one-year mortality in subjects with a higher number of glycemic fluctuations during hospitalization [39].

Continuing with our analyses, a second logistic regression model, which substituted the variable intra-hospital CV ≥ 25.8% for SD, showed results consistent with those recorded in the first regression. Specifically, after hemiplegia and dementia, both sex and length of stay were strongly predictive (OR 8.64 and 0.86, respectively), with intra-hospital glycemic CV ≥ 25.8% following closely with an OR of 7.53 (*p* = 0.028).

It is significant that there is only one comment concerning this last variable: as seen, the data about glycemic variability expressed as SD could have a role in determining hospitalization outcomes, and the glycemic CV, which also provides information regarding the mean glucose levels recorded within a time frame, cannot be any less important. This has been suggested by recent observations in a home setting and allows the CV to play a key role in the extra-hospital glycemic monitoring of diabetic subjects. In particular, a 2019 consensus, also adopted as a model by the Italian Society of Endocrinology (SIE) [40], established that a CV < 33% is able to minimize home hyper- and hypoglycemic events, as well as protect diabetic subjects from chronic complications of T2DM, especially microvascular ones, as highlighted in a study by Hirsch et al. back in 2015 [41].

A significant degree of predictability for 30-day mortality from hospital admission is also attributed to the variable our research group introduced in this study (estimated pre-hospitalization glycemic CV), which was introduced in the third logistic regression analysis alongside the previously described variables. In terms of predictive strength, this index appeared after hemiplegia, dementia, and sex, showing an OR of 4.84 (*p* = 0.042), and showed that the relationship between intra-hospital glycemic variability and average pre-hospitalization blood glucose levels (derived from HbA_1c_) is closely associated with 30-day all-cause mortality in patients with T2DM.

It should be noted that, in all three analyses, the length of stay was the only variable that presented a “protective” effect against 30-day mortality. However, this data has been previously discussed and does not deserve further comments.

To provide a more in-depth estimate of the effectiveness of the variables under consideration, three different Cox regression analyses were conducted on our population of diabetic patients, adjusting for age, sex, and comorbidities (using the CCI). These analyses showed that, at 30 days, all subgroups of patients with worse glycemic control (values of SD, intra-hospital glycemic CV, and estimated pre-hospitalization glycemic CV above the calculated cut-off) had lower survival rates; however, for the variable intra-hospital glycemic CV it can be observed that the survival curves of subjects with values below and above the 25.8% cut-off intersected in the first days of hospitalization, indicating a poor discriminatory capacity between populations with better or worse outcomes, at least for that specific period of stay.

As a whole, the study seems to confirm both known aspects in the literature, such as the association between high glycemic fluctuations and increased 30-day mortality in patients with a diagnosis of T2DM, and it reveals a completely original aspect that we believe might be considered during the initial in-hospital evaluation of these subjects. The association between average blood glucose levels, both during hospitalization and pre-hospitalization (the latter easily obtainable through HbA_1c_ measurement), and the degree of intra-hospital glycemic variability seems to have a high capacity to discriminate patients at a higher risk of dying within 30 days from hospital admission for all causes. Pre-hospitalization average blood glucose, when compared with intra-hospital glycemic variability (placed in the denominator), shows an even stronger association than what was calculated during hospitalization, suggesting, if it is still needed, that measuring HbA_1c_ is useful and necessary for the correct evaluation of diabetic patients.

The limitations of this study are numerous, primarily due to its retrospective nature, which does not allow for any interventional changes to be made to the patients during the course of the study. It should be emphasized, as previously mentioned, that no information regarding the reasons for hospital admission for the patients has been reported (only surgical causes were excluded), nor information about the treatments administered to them during hospitalization.

The small sample size (120 patients, calculated based on the percentage of diabetic patients hospitalized per month in our department) and the single-center nature of the study do not allow for any definitive conclusions to be drawn about the observations made. Furthermore, the absence of continuous glucose monitoring (or flash monitoring) in favour of point-of-care testing (POCT) certainly does not provide the same level of precision in glycemic sampling and is influenced by the methodology in use.

We are aware, therefore, that a significant expansion of the analytical sample is necessary to overcome, at least in part, the limitations described. Similarly, the definition of a standardized research protocol and the involvement of a larger number of participating centers could lead to significant discoveries in this area of in-hospital management of diabetic patients, which is still largely unexplored.

## Conclusions

While the measurement of HbA_1c_ remains the gold standard for regular monitoring of diabetic individuals, glycemic control within and outside hospital settings should target the mitigation of daily and periodic fluctuations. Excessive fluctuations in blood glucose levels (i.e., high glycemic variability, as indicated by the standard deviation, SD) represent an additional risk factor for mortality in these individuals. Within our population, this was demonstrated as early as the 30^th^ day from hospital admission for non-surgical causes.

In conclusion, our study proposes that the ratio between intra-hospital glycemic variability and pre-hospitalization glycemic mean (derived from circulating levels of HbA_1c_) could serve as an additional valuable index for glycemic control in patients diagnosed with T2DM, even during hospitalization.

### Electronic supplementary material

Below is the link to the electronic supplementary material.

Supplementary Material 1: Table 1 HbA_1c_, glycosylated haemoglobin; CKD, chronic kidney disease; TIA, transient ischemic attack; PAD, peripheral arterial disease; COPD, chronic obstructive pulmonary disease; T2DM, type 2 diabetes mellitus; DPP-4is, dipeptidyl peptidase 4 inhibitors; GLP-1 RAs, glucagon-like peptide 1 receptor agonists; SGLT-2is, sodium-glucose co-transporter 2 inhibitors. Fig. 1 Design of the study. I/E = Inclusion/Exclusion; SD = standard deviation; CV = coefficient of variation.

## Data Availability

No datasets were generated or analysed during the current study.

## Abbreviations

T2DM: Type 2 Diabetes Mellitus
HbA_1c_: Glycated Hemoglobin
ML: Machine Learning
CCI: Charlson Comorbidity Index
STROBE: Strengthening the Reporting of Observational studies in Epidemiology
SD: Standard Deviation
FAS: Feature Augmentation and Selection
ROC: Receiver Operating Characteristic
CV: Coefficient of Variation
OR: Odds Ratio
CI: Confidence Intervals
AUC: Area Under Curve
SGLT-2: Sodium Glucose Cotransporter 2
TIR: Time In Range
TAR: Time Above Range
TBR: Time Below Range
AGP: Ambulatory Glucose Profile
MAGE: Mean Amplitude of Glycemic Excursions
CGM: Continuous Glucose Monitoring
FGM: Flash Glucose Monitoring
