# Supplementary material for: Pre-hospital glycemia as a biomarker for in-hospital all-cause mortality in diabetic patients - a pilot study
Source: Cardiovasc Diabetol. 2024 May 3;23:153. doi: 10.1186/s12933-024-02245-8 (PMC11069282; doi:10.1186/s12933-024-02245-8)
Supplement: Supplementary file 1 — Supplementary Material 1: Table 1 HbA1c, glycosylated haemoglobin; CKD, chronic kidney disease; TIA, transient ischemic attack; PAD, peripheral arterial disease; COPD, chronic obstructive pulmonary disease; T2DM, type 2 diabetes mellitus; DPP-4is, dipeptidyl peptidase 4 inhibitors; GLP-1 RAs, glucagon-like peptide 1 receptor agonists; SGLT-2is, sodium-glucose co-transporter 2 inhibitors. Fig. 1 Design of the study. I/E = Inclusion/Exclusion; SD = standard deviation; CV = coefficient of variation. [file 12933_2024_2245_MOESM1_ESM.docx]

**Supplementary Information**

**Appendix**

1. **Characteristics of the training and test splits**

In this section, we report the characteristics of the training and test population we used to develop and evaluate the machine-learning-based classifiers.

| Personal informations | Train set (n=90) | Test set (n=30) | *p* value |
| --- | --- | --- | --- |
| Age, years±SD | 78.0±9.0 | 79.6±8.5 | 0.41 |
| Days of stay, n | 14.5±11.9 | 14.7±12.5 | 0.88 |
| Females, n (%) | 33 (36.7) | 15 (50.0) | 0.20 |
| Males, n (%) | 57 (63.3) | 15 (50.0) |  |
| Smoke habit, n (%) | 32 (35.6) | 12 (40.0) | 0.66 |
| Glycaemic informations |  |  |  |
| HbA_1c_ (%) | 7.0±1.8 | 6.7±0.9 | 0.13 |
| pre-hospital blood glucose, mean±SD | 155.4±51.5 | 145.3±27.1 | 0.14 |
| in-hospital blood glucose, mean±SD | 163.9±38.9 | 173.0±47.5 | 0.30 |
| in-hospital median blood glucose, mean±SD | 156.7±39.3 | 165.5±45.0 | 0.31 |
| in-hospital/pre-hospital average blood glucose ratio, mean±SD | 0.96±0.06 | 0.96±0.04 | 0.51 |
| glycemic variability, mean±SD | 46.4±20.4 | 48.5±21.9 | 0.64 |
| Comorbidities |  |  |  |
| Hypertension, n (%) | 70 (77.8) | 22 (73.3) | 0.62 |
| Ischemic heart disease, n (%) | 24 (26.7) | 4 (13.3) | 0.14 |
| Heart failure, n (%) | 10 (11.1) | 6 (20.0) | 0.22 |
| Moderate or severe CKD, n (%) | 24 (26.7) | 8 (26.6) | 1.00 |
| Stroke or TIA, n (%) | 15 (16.7) | 4 (13.3) | 0.67 |
| PAD, n (%) | 9 (10.0) | 5 (16.7) | 0.33 |
| COPD, n (%) | 21 (23.3) | 10 (33.3) | 0.28 |
| Mild hepatopathy, n (%) | 2 (2.2) | 0 (0.0) | 0.41 |
| Severe hepatopathy, n (%) | 1 (1.1) | 0 (0.0) | 0.56 |
| Peptic ulcer disease, n (%) | 0 (0.0) | 0 (0.0) | - |
| AIDS, n (%) | 0 (0.0) | 0 (0.0) | - |
| Hemiplegia, n (%) | 4 (4.4) | 1 (3.3) | 0.79 |
| Localized or hematological malignancy, n (%) | 17 (18.9) | 10 (33.3) | 0.10 |
| Metastatic malignancy, n (%) | 5 (5.6) | 2 (6.6) | 0.82 |
| Dementia, n (%) | 23 (25.6) | 8 (26.6) | 0.90 |
| Rheumatological disease, n (%) | 2 (2.2) | 0 (0.0) | 0.41 |
| T2DM without chronic complications, n (%) | 41 (45.6) | 15 (50.0) | 0.67 |
| T2DM with chronic complications, n (%) | 49 (54.4) | 15 (50.0) |  |
| CCI, mean±SD | 4.1±2.3 | 4.4±2.7 | 0.54 |
| Antidiabetic treatment |  |  |  |
| Diet only, n (%) | 11 (12.2) | 3 (10.0) | 0.74 |
| Metformin only, n (%) | 22 (24.4) | 9 (30.0) | 0.55 |
| Sulphonylureas only, n (%) | 2 (2.2) | 0 (0.0) | 0.41 |
| DPP-4is only, n (%) | 3 (3.3) | 1 (3.3) | 1.00 |
| GLP-1 RAs only, n (%) | 0 (0.0) | 1 (3.3) | 0.08 |
| Insulin only, n (%) | 19 (21.1) | 7 (23.3) | 0.80 |
| SGLT-2is only, n (%) | 3 (3.3) | 2 (6.7) | 0.43 |
| Other drugs or combinations of drugs, n (%) | 29 (32.2) | 7 (23.3) | 0.36 |

**Supplementary Table 1** HbA_1c_, glycosylated haemoglobin; CKD, chronic kidney disease; TIA, transient ischemic attack; PAD, peripheral arterial disease; COPD, chronic obstructive pulmonary disease; T2DM, type 2 diabetes mellitus; DPP-4is, dipeptidyl peptidase 4 inhibitors; GLP-1 RAs, glucagon-like peptide 1 receptor agonists; SGLT-2is, sodium-glucose co-transporter 2 inhibitors

1. **Comparison with other strong baseline models**

Given the small amount of data, in this work, we chose to pay special attention to feature engineering. We thus developed a new feature engineering method called Feature Augmentation and Selection (FAS), which proved to be very effective in boosting the performance of the AdaBoost classifier (see Figure 2). The final trained model, AdaBoost-FAS, showed satisfactory performance: it was able to classify our test population with a balanced accuracy of 0.856. How does this model compare to off-the-shelf hyperparameter-tuned classifiers? To address this question, we considered four additional classifiers: Support Vector Machine (SVM), eXtreme Gradient Boosting (XGBoost), Light Gradient Boosting Machine (LightGBM), and Random Forest (RForest). All classifiers were trained using the SMOTE-augmented training set and tested on the independent test set. For each model, we performed hyperparameter tuning with the Optuna library by sampling with the Tree-structured Parzen Estimator (TPE) algorithm for 500 trials. Figure 5 summarizes the results of this analysis: the best model resulting from this analysis is RForest, which reached a test balanced accuracy of 0.788, which is lower than the one reached by our model, AdaBoost-FAS (0.856). This analysis confirms that careful feature engineering procedures such as FAS are very valuable in the low-data regime.


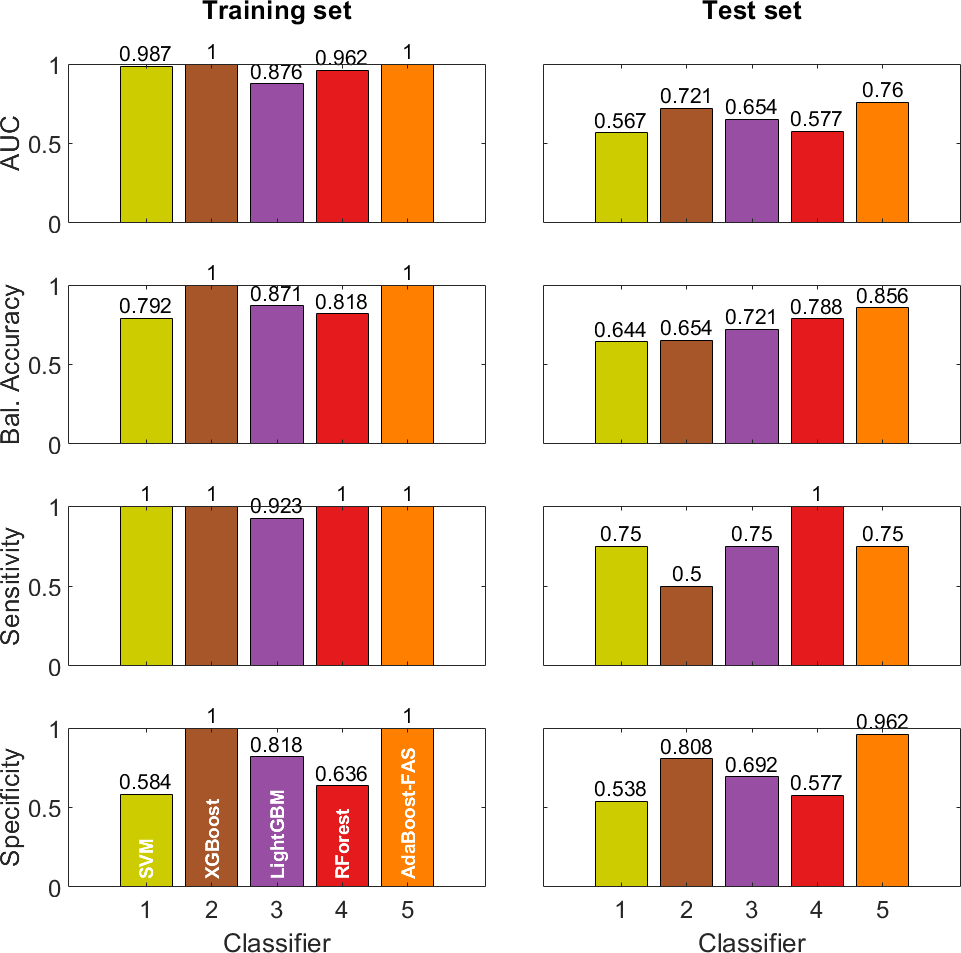


**Figure 5** Comparison of AdaBoost-FAS with other strong baseline models.


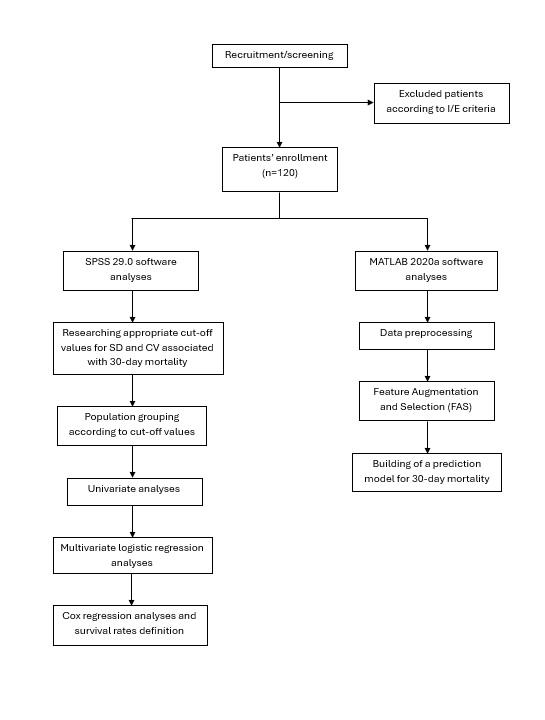


**Supplementary Figure 1** Design of the study. I/E= Inclusion/Exclusion; SD=standard deviation; CV= coefficient of variation
